# Intracellular Localization and Conformational State of Transglutaminase 2: Implications for Cell Death

**DOI:** 10.1371/journal.pone.0006123

**Published:** 2009-07-01

**Authors:** Soner Gundemir, Gail V. W. Johnson

**Affiliations:** 1 Department of Pharmacology and Physiology, University of Rochester, Rochester, New York, United States of America; 2 Department of Anesthesiology, University of Rochester, Rochester, New York, United States of America; University of Texas MD Anderson Cancer Center, United States of America

## Abstract

Transglutaminase 2 (TG2) is a multifunctional enzyme that has guanine nucleotide binding and GTP hydrolyzing activity in addition to its transamidating function. Studies show that TG2 is a player in mediating cell death processes. However, there is far from a consensus about the role of this enzyme in cell death processes as it appears to be dependent upon the cell type, stimuli, subcellular localization and conformational state of the enzyme. The purpose of this study was to dissect the role of TG2 in the cell death processes. To this end, we created and characterized 4 distinct point mutants of TG2, each of which differs from the wild type by its conformation or by lacking an important function. We also prepared these mutants as nuclear targeted proteins. By overexpressing mutant or wild type forms of TG2 in HEK 293 cells, we investigated the modulatory role of the protein in the cell death process in response to three stressors: thapsigargin, hyperosmotic stress and oxygen/glucose deprivation (OGD). All of the TG2 constructs, except the R580A mutant (which cannot bind guanine nucleotides and is therefore more prone to exhibit transamidating activity), either did not significantly affect the cell death processes or were protective. However in the case of the R580A mutant, cell death in response to high thapsigargin concentrations, was significantly increased. Intriguingly, nuclear localization of R580A-TG2 was sufficient to counteract the pro-death role of cytoplasmic R580A-TG2. In addition, nuclear localization of TG2 significantly facilitated its protective role against OGD. Our data support the hypothesis that the transamidation activity of TG2, which is mostly quiescent except in extreme stress conditions, is necessary for its pro-death role. In addition, nuclear localization of TG2 generally plays a key role in its protective function against cell death processes, either counteracting the detrimental effect or strengthening the protective role of the protein.

## Introduction

Transglutaminase 2 (TG2, EC 2.3.2.13) is a member of the transglutaminase (TG) family that catalyzes thiol- and Ca^2+^-dependent transamidation reactions. The transamidation reaction that is catalyzed by the TG family is the formation of a covalent bond between the γ-carboxamide group of a peptide bond glutamine residue and a primary amine group [1]. TG2 is the most ubiquitously expressed and is the most studied member of the family. In addition to its transamidating activity, it binds and hydrolyzes GTP [2]. Ca^2+^ and guanine nucleotide binding inversely regulate the transamidating activity of TG2; that is, TG2 is only active as a transglutaminase when bound to Ca^2+^ and inactive when bound to the guanine nucleotides [3]. However, in the guanine nucleotide bound form, TG2 is proposed to be involved in regulating signal transduction by acting as a G-protein which transduces a signal from several different receptors to phospholipase C-δ1 [4]. Moreover, recent developments shows it truly is a multifunctional protein as it is demonstrated to function as a protein disulfide isomerase (PDI) [5] and protein kinase [6], [7]. Lastly, it has been shown to interact with certain proteins with no clear evidence of enzymatic activity playing a role, which indicates that it also can act as a protein scaffold [8], [9].

The structural basis for its multifunctionality is partly clear since the crystal structure of TG2 is available both in its GDP bound closed conformation and in an inhibitor bound open conformation [10], [11]. However, none of the crystals include bound Ca^2+^; therefore Ca^2+^-binding residues are not known with certainty, although structure prediction and site directed mutagenesis studies have suggested at least 3 different Ca^2+^-binding sites [12], [13]. The prevailing view is that the binding of the Ca^2+^ ions and guanine nucleotides have opposite effects on the transamidating function of the enzyme; the former potentiates this function while the latter attenuates it [3], [14]. However, TG2 has a much higher affinity for the guanine nucleotides than it has for Ca^2+^ [12]. Therefore, it has been proposed that in the cell, the transamidating activity of TG2 is usually latent and only is expressed during processes such as apoptosis or differentiation. This view is further strengthened by the recent observation that even extracellular TG2 is not active as a transamidating enzyme in normal physiological conditions, although it is activated during wound healing [15], which represents a more pathological condition. However, previous studies have indicated that the transamidating activity of extracellular TG2 might be important for the normal physiological processes such as extracellular matrix stabilization [16], [17]. These seemingly controversial observations on the activity state of extracellular TG2 suggest that there are other, yet unidentified, factors that can regulate transamidating function of TG2 in addition to Ca^2+^ ions and the guanine nucleotides [15].

The catalytic site of transamidating activity is composed of the catalytic triad characteristic of cysteine proteases; cysteine 277 (C277), histidine 335 (H335) and aspartate 358 (D358) are the critical residues for transamidating activity in human TG2 [10]. The cysteine to serine mutation at the position 277 (C277S) has been extensively used to inactivate the transamidation function of TG2 [18], [19]. Although it knocks out all transamidating activity, it also results in a conformation change which greatly impairs the guanine nucleotide binding capability as well [10], [20]. In addition to the catalytic triad, a conserved tryptophan residue (W241) is also critical for the transamidating activity and mutating this residue to an alanine (W241A) knocks down all transamidating activity without any effect on guanine nucleotide binding [21]. The guanine nucleotide binding pocket, on the other hand, is composed of at least ten residues; however, one arginine residue (R580) interacts with the guanine nucleotide at several points and its mutation to alanine (R580A) results in the almost complete loss of guanine nucleotide binding activity without any significant effect on the transamidating activity [20], [22]. Finally, TG2 is proposed to have open and closed conformers, with notably distinct features [3], [11]. The closed form is used as an equivalent to the GTP/GDP bound form, which is predicted to be more compact than the open conformer. The tyrosine residue at the position 516 (Y516) is critical to attaining the closed conformation and mutation of this residue to phenylalanine (Y516F) renders TG2 more prone to an open conformation [3], [11].

A primary role of TG2 in the cell is to modulate cell death processes. However the function of TG2 in cell death is complex and context specific. Depending on the stressor and cell type, TG2 can either facilitate or ameliorate cell death processes [12], [18], [22], [23], [24], [25], [26], [27]. This complexity may be due to its different activities, cellular localization, different isoforms or different conformations [18], [28], [29], [30]. There are studies suggesting that transamidating activity is required for its protective role [31], as well as the studies showing that transamidating activity actually facilitates apoptosis [18]. There is substantial evidence suggesting that guanine nucleotide binding renders TG2 more protective [22]. However, there is controversy as to whether the protection is due to guanine nucleotide binding *per se* or suppressed transamidating activity as a result of this binding, an issue that remains unresolved.

In this study we investigated the TG2 – cell death relationship focusing on three of these variables: i) its conformation/activity state, ii) localization (nuclear or cytoplasmic) and iii) the type of stressor used to induce cell death. This was accomplished by generating 4 different point mutations each of which has a unique effect on the conformation/activity state of the protein with and without a nuclear localization signal (NLS) and measuring how they modulate cell death in response to three distinct stressors: hyperosmotic stress, thapsigargin and oxygen/glucose deprivation. In our models the transamidating activity of TG2 was very low under resting conditions; and as long as the protein was silent in terms of transamidating activity, it was either neutral or protective against cell death. However, the R580A mutation which resulted in an increase in *in situ* transamidating activity also facilitated TG2's role as a pro-death protein. Nuclear localization of TG2, especially when it was not active as a transamidating enzyme, almost invariably resulted in an attenuation of cell death. The Y516F mutation causes similar changes in the guanine nucleotide binding and transamidating activity characteristics of the protein as R580A, although the latter mutation is a considerably more potent mutation than the former. Very intriguingly, for certain stressors, the cell death profile of Y516F mutant was similar to that of R580A. Overall, this study strongly suggests that the major function of TG2 in the cell does not involve its transamidating activity and that it attenuates cell death in a transamidating activity independent manner when localized to the nucleus.

## Results

### Expression of TG2 constructs in HEK 293A cells

**Figure 1:**
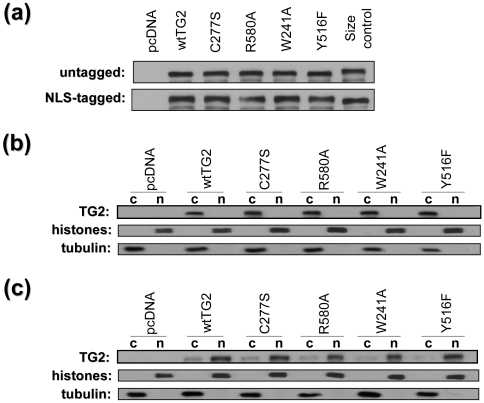
Expression and Subcellular Localization of TG2 constructs. Representative immunoblots of cell lysates from HEK 293A cells which were transiently transfected with TG2 constructs. (a) Expression of TG constructs. Lysates were blotted for TG2. untagged: TG2s *without* an NLS tag, except size control (which is NLS-tagged wild type). NLS-tagged: TG2s *with* NLS tag, except lane size control (which is untagged wild type). (b) Subcellular localization of untagged TG2 variants. Twenty four hours posttransfection cells were separated into nuclear and cytosolic fractions and were blotted for TG2, histones and tubulin proteins. Histone (nuclear marker) and tubulin (cytosolic marker) blots show the purity of the fractions. TG2 blot shows that untagged TG2 variants are localized almost exclusively to the cytosol and none of the mutations has a significant effect on the localization of TG2. (c) Subcellular localization of NLS-tagged TG2 variants. Twenty four hours posttransfection cells were separated into nuclear and cytosolic fractions and were blotted for TG2, histones and tubulin proteins. TG2 blot shows that more than 80% of the total amount of NLS-tagged TG2 variants are localized to the nucleus and none of the mutations has a significant effect on the localization of NLS-TG2. 10 µg of protein was loaded in each well.

The relative expression levels of the different TG2 constructs were determined by immunoblotting. To equalize expression levels of the TG2 the amount of transfected plasmid DNA was adjusted for each construct (Fig. 1). The untagged TG2 constructs migrated at approximately 77 kDa whereas the NLS/myc tag decreased the electrophoretic mobility of the protein (Fig. 1a).

### Subcellular localization of TG2 in transfected cells by different TG2 constructs

The subcellular localization of the untagged and NLS-tagged TG2 constructs was determined by separating transfected cells into cytosolic and nuclear fractions. The purity of cytosolic and nuclear fractions was demonstrated by immunoblotting for histones (nuclear proteins) and α-tubulin (a cytosolic protein) (Fig. 1b&c). As expected, only trace amounts of TG2 were found in the nucleus with the untagged TG2 transfected cells (Fig. 1b), as it has previously been shown that endogenous or exogenous TG2 is mainly cytoplasmic [32]. The NLS tagged TG2 constructs, however, were found by densitometric analysis predominantly in the nucleus (approximately 80%) (Fig. 1c). Notably, none of the mutations significantly changed the subcellular localization of TG2 (Fig. 1b&c).

### The effect of the mutations on the affinity of TG2 for guanine nucleotides

**Figure 2:**
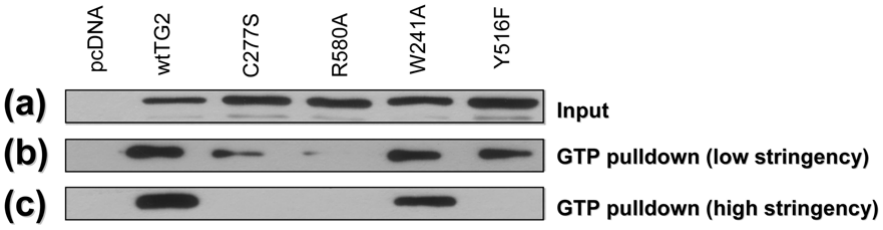
GTP Binding of TG2 Mutants. Immunoblot showing GTP binding of different TG2 constructs. (a) expression levels of TG2 constructs. 10 µg of protein was loaded in each well. Pulldown experiments were performed with GTP agarose using low (b) and high (c) stringency conditions to evaluate the strength of binding between TG2 and GTP.

To determine the effect of the mutations on TG2, a GTP agarose pull down assay was used [33]. The cells were transfected with the untagged TG2 constructs and collected 24 h later. In order to evaluate the relative affinities of the TG2 constructs for GTP, we used two different stringency conditions for this assay. In the low stringency conditions we used 150 mM NaCl and 0.1% Triton-X and in the high stringency conditions we used 300 mM NaCl and 0.5% Triton-X. At high stringency, only wild type TG2 and W241A-TG2 effectively bound to the GTP-agarose; which demonstrates that the W241A mutation has no effect on the guanine nucleotide binding ability of the protein (Fig. 2). However, at low stringency, in addition to wild type and W241A-TG2; Y516F-TG2 and, to a much lesser extent, C277S-TG2 was found to bind to GTP agarose (Fig. 2). The results from the GTP-agarose pull down assay suggest that the guanine nucleotide binding affinities of TG2 constructs are as follows: wild type = W241A>Y516F>C277S>>R580A. These findings are in agreement with previous studies [3], [20], [34].

### The effect of the mutations on the in situ transamidating activity of TG2

**Figure 3:**
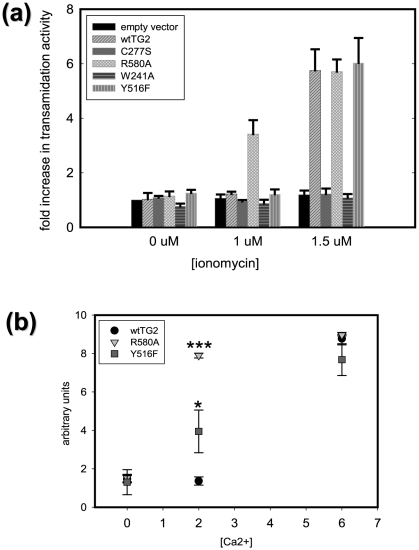
Transamidating activity of TG2 variants. Graphs showing *in situ* (a) and *in vitro* (b) transamidating activities of different TG2 mutants as a function of ionomycin concentration (µM) in the cell culture medium (a) or calcium ion added (mM) to the reaction mixture (b) (N = 3). Results are shown as mean+/−SE *p<0.05, **p<0.01, ***p<0.005.

*In situ* transamidating activities of the untagged TG2 constructs were determined. Twenty-four hours post-transfection no transamidating activity was detected in basal conditions for any of the TG2 constructs. Ionomycin was used to increase intracellular Ca^2+^ concentrations and activate TG2. In the presence of 1 µM ionomycin an approximately 3 fold increase was observed in the transamidating activity of R580A-TG2 transfected cells (Fig. 3a). This result is as expected as this mutation significantly decreases the affinity of TG2 for guanine nucleotides (Fig. 2), the most well known intracellular TG2 inhibitors [34]. At an ionomycin concentration of 1.5 µM, wild type TG2 and Y516-TG2 transfected cells also exhibited transamidating activity. At this concentration of ionomycin wild type, R580A-TG2 and Y516F-TG2 transfected cells all exhibited *in situ* transamidating activity that was approximately 6 fold over vector control (Fig. 3a). No transamidating activity was detected with the W241A and C277S mutations as expected (Fig. 3a).

### The effect of the mutations on the in vitro transamidating activity of TG2

To evaluate total transamidating activity, in vitro transamidating activities were measured in lysates collected from the cells transfected with each of the untagged TG2 constructs. In this assay, increasing concentrations of Ca^2+^ were used to determine the relative activation state of each of the TG2 constructs. *In vitro* transamidating activities of wild type TG2, R580A-TG2 and Y516F-TG2 were expressed as a fold increase over vector control lysates. The assay was conducted at various Ca^2+^ concentrations in the range of 0–10 mM, but only the data for 0, 2 and 6 mM added Ca^2+^ are shown (Fig. 3b). The activities of W241A-TG2 and C277S-TG2 are not shown since they exhibited no detectable *in vitro* transamidating activity. The results from the *in vitro* transamidating activity measures were similar to those of the ones obtained with *in situ* transamidating assay (Fig. 3b). In the absence of any exogenously added Ca^2+^, there was no detectable activity. Addition of 2 mM Ca^2+^ was sufficient to fully activate R580A-TG2 (approximately 8 fold over vector control) and increase transamidating activity in the lysates of Y516F-TG2 transfected cells approximately 3 fold over vector control. However at 2 mM Ca^2+^, wild type-TG2 exhibited no detectable activity. The addition of 6 mM exogenous Ca^2+^ was sufficient to fully activate the transamidating activity (approximately 8 fold over vector control) for all of TG2 constructs that were catalytically active: wild type, R580A-TG2 and Y516F-TG2. These results indicate that the Y516F mutation compromises guanine nucleotide binding which results in a lower Ca^2+^ threshold for the activation of transamidating activity compared to wild type TG2.

### Cytosolic R580A-TG2 promotes thapsigargin-induced toxicity in HEK 293A cells

**Figure 4:**
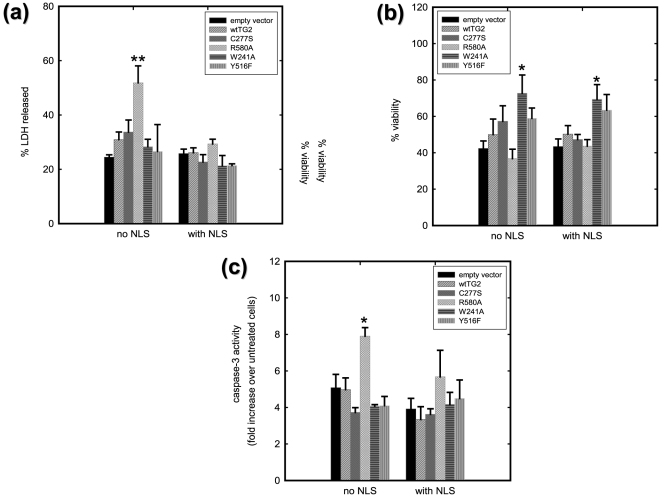
TG2 can either protect against or facilitate thapsigargin induced cell death in HEK 293A cells depending on its conformation and its localization. (a) LDH release after 9 h of 15 µM thapsigargin treatment. LDH release is significantly increased in HEK 293A cells which express R580A-TG2 without an NLS tag. (N = 3). (b) Cell viability determined by the resazurin assay after 10 h of 15 µM thapsigargin treatment. Metabolic activity is significantly increased in HEK 293A cell which express W241A-TG2 with or without an NLS tag. (N = 4) (c) Caspase-3 activity after 6 h of 15 µM thapsigargin treatment. Caspase-3 activity is significantly increased in HEK 293A cells which express R580A without an NLS tag. (N = 3). Results are shown as mean+/−SE *p<0.05, **p<0.01.

HEK 293A cells were transfected with the TG2 constructs and 24 h later they were treated with 15 µM thapsigargin. The effect of thapsigargin on cell viability was determined using three different measures; LDH release, a resazurin to resorufin conversion assay and measurement of caspase-3 activity. After 9 h of thapsigargin treatment, approximately 25% of total LDH was released to the media in vector control cells (Fig. 4a). Untagged R580A-TG2 significantly increased LDH release to approximately 50% of the total LDH (Fig. 4a). The toxic effect of the untagged R580A-TG2 was confirmed by the caspase-3 assay, as well. After 4 h of thapsigargin treatment, a 4–5 fold increase in caspase-3 activity was observed in vector control cells and an approximately 8 fold increase in untagged R580A-TG2 transfected cells (Fig. 4c). These findings are particularly interesting given that thapsigargin treatment of R580A-TG2 transfected cells results in a slight but significant increase in *in situ* transamidating activity, while this is not the case for the other TG2 constructs (data not shown, see discussion). These results suggest that the increase in the transamidating activity of R580A-TG2 in response to thapsigargin treatment may result in facilitation of cell death in a caspase-dependent manner. The resazurin assay, which measures the reducing potential of the cell, revealed no significant differences between R580A-TG2 transfected cells and vector control cells, in response to thapsigargin (Fig. 4b). Interestingly, both untagged and NLS-tagged W241A-TG2 significantly attenuated the loss of reducing potential in response to thapsigargin compared to vector cells as measured by resazurin to resorufin conversion (Fig. 4b). These results indicate that depending on the conformation and activity state, TG2 can differentially affect distinct aspects of cell death processes.

### Cytosolic W241A-TG2 protects against serum starvation and thapsigargin-induced toxicity in HEK 293TN cells

**Figure 5:**
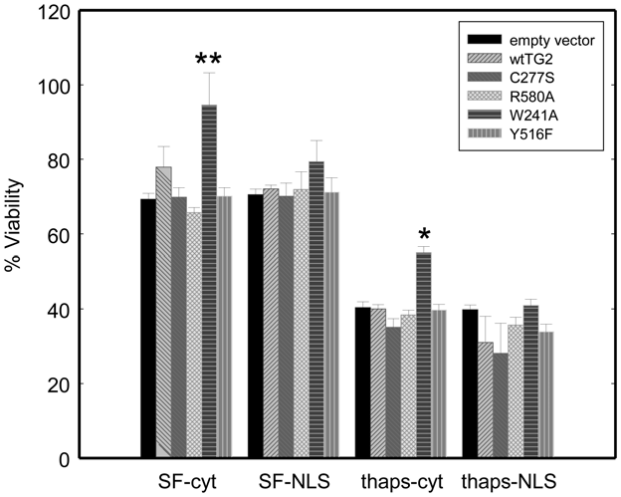
Cytosolic W241A-TG2 protects against serum starvation and thapsigargin-induced toxicity in HEK 293TN cells. Cell viability determined by the resazurin assay after 24 h of serum starvation and serum starvation combined with 2.5 µM thapsigargin treatment. Cytosolic W241A-TG2 significantly improves metabolic activity of HEK 293TN cell both after serum starvation alone and serum starvation combined with thapsigargin treatment. (N = 3) Results are shown as mean+/−SE *p<0.05, **p<0.01.

To examine the effect of TG2 on cell viability in the absence of pronounced cell death, HEK 293TN cells were transfected with the TG2 constructs and 24 h later they were transferred to serum free media and incubated with or without 2.5 µM thapsigargin. After 24 h of serum starvation alone or serum starvation combined with thapsigargin treatment, cell viability was measured using the resazurin assay. Serum starvation decreased the cell viability to ∼70% in vector control cells compared to non-serum-starved cells. Cytoplasmic W241A-TG2 mutant almost completely counteracted the effect of serum starvation and increased the viability to ∼95% (Fig. 5). Serum starvation combined with thapsigargin had a more severe impact on cell survival and decreased the viability to ∼40%. Yet again, cytoplasmic W241A-TG2 construct significantly protected against serum starvation combined with thapsigargin treatment and increased the viability to ∼55% (Fig. 5) No increase in LDH release assay or caspase-3 activity were observed after 24 h of 2.5 μM thapsigargin treatment and/or serum starvation (data not shown).

### TG2 protects HEK 293A cells against hyperosmotic stress in a caspase-independent manner, regardless of its conformation and intracellular localization

**Figure 6:**
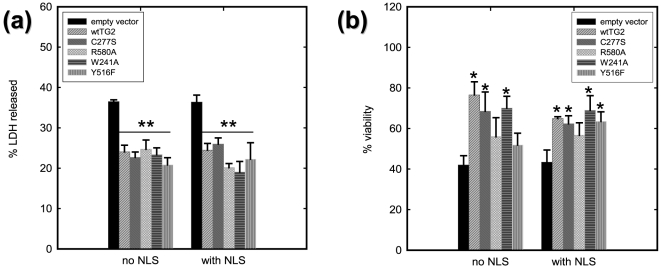
TG2 protects HEK 293A cells from hyperosmotic stress, independent of its localization and conformation. (a) LDH release after 10 h of 1.0 M sorbitol treatment. LDH release is significantly decreased in HEK 293A cells which express TG2 regardless of the conformation state or its subcellular localization (N = 5). (b) Cell viability determined by the resazurin assay after 6 h of 1.0 M sorbitol treatment. Hyperosmotic stress-induced decreases in metabolic activity are significantly attenuated by TG2 constructs except for untargetted R580A-TG2, untargetted Y516F-TG2 and nuclear targetted R580A-TG2 (N = 4). Results are shown as mean+/-SE *p<0.05, **p<0.01.

After 3–4 h of 1.0 M sorbitol treatment all cells started to show signs of apoptosis such as membrane blebbing (data not shown). 10 h of 1.0 M sorbitol treatment resulted in approximately 35% LDH release in vector control cells. Overexpression of any of the TG2 constructs, regardless of nuclear or cytoplasmic localization, significantly decreased LDH release (Fig. 6a). The results of resazurin assay, which was performed after 6 h of sorbitol treatment, were similar to LDH release assay results, however, the variance in the data between the constructs was more pronounced. The results from resazurin assay show that, hyperosmotic stress decreased the cell viability to ∼40% in vector control cells. Most of the TG2 constructs caused a significant improvement in the viability (up to 60%–80%). However, cytosolic R580A-TG2, cytosolic Y516F-TG2 and nuclear R580A-TG2 constructs did not increase cell viability compared to vector cells (Fig. 6b). Interestingly, the protective effect of TG2 against hyperosmotic stress was not due to a decrease in caspase-3 activity. There was an approximately 3.5 fold increase in the caspase-3 activity after 6 h of sorbitol treatment compared to isosmotic conditions in cells transfected with the TG2 constructs as well as the empty vector (data not shown).

### Nuclear localized and catalytically quiescent TG2 protects HEK 293A cells against oxygen/glucose deprivation (OGD) induced cell death

**Figure 7:**
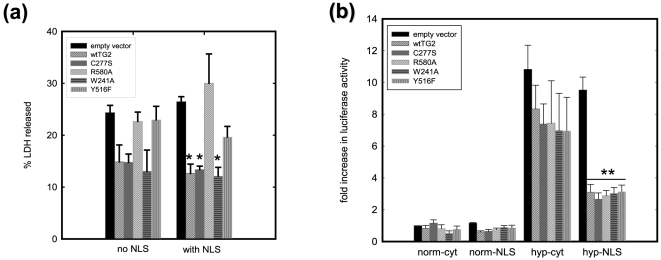
Nuclear targeted and transamidating inactive TG2 protects HEK 293A cells from oxygen/glucose deprivation triggered cell death independent of HIF signaling. (a) LDH release after 16 h of oxygen/glucose deprivation (OGD). LDH release is significantly decreased in HEK 293A cells which express wild type-TG2, C277S-TG2 or W241A-TG2 with an NLS tag upon oxygen/glucose deprivation. (N = 5) (b) HRE luciferase activity after 16 h of 0.1% oxygen treatment. All nuclear targeted TG2 variants significantly decreased hypoxia responsive gene transcription whereas none of the untargeted TG2 variants had a significant effect. Results are shown as mean+/−SE *p<0.05, **p<0.01.

To induce oxygen/glucose deprivation (OGD), cells were maintained at 0.1% oxygen in serum and glucose free media for 16 h of OGD which resulted in approximately 25% LDH release in vector control cells (Fig. 7a). LDH release was significantly attenuated in NLS-wild type-TG2, NLS-C277S-TG2 and NLS-W241-TG2 transfected cells (Fig. 7a). No increase in caspase-3 activity was detected in this OGD paradigm (data not shown), and the resazurin assay could not be used in this stress paradigm.

We have recently reported that TG2 interacts with hypoxia inducible factor 1β (HIF1β), attenuates HIF signaling and protects against OGD induced cell death in a different cell model [9]. Therefore we transfected an HRE reporter plasmid together with the TG2 constructs to monitor HIF activity. The results shown in Fig. 7b demonstrates that all NLS-tagged TG2 constructs, but not their untagged counterparts, significantly suppressed HIF-dependent transcription after 16 h of hypoxia. An NLS-tagged GFP construct was used as a negative control to test whether overexpressing a nonspecific protein in the nucleus was sufficient to suppress HIF-dependent transcription. As expected, nuclear localized GFP had no significant effect on HIF activity (data not shown). In summary, although all of the nuclear TG2 constructs attenuated HIF signaling, only the catalytically silent TG2 variants (including wild type TG2 which has very low transamidating activity except in excessive stress conditions) protected cells against OGD-induced cell death.

## Discussion

In order to understand the differential roles of TG2 in cell death processes, we examined how TG2 regulates cell death in response to 3 different cell stressors: hyperosmotic stress, thapsigargin and OGD. In addition, we created TG2 mutants that mimic different activity and conformation states of TG2 that were either untagged or targeted specifically to the nucleus.

The transamidating activity and GTP binding behavior of the constructs was as would be predicted based on our knowledge of the structure and function of TG2 [3], [10], [11]. However, one interesting observation was that transient transfection of R580A-TG2 did not result in an increase in basal transamidating activity, although increased *in situ* transamidating activity was observed in response to ionomycin. Since this mutation almost abolishes guanine nucleotide binding activity — the most well known intracellular TG2 inhibitors — basal increases in transamidating activity in R580A-TG2 transfected cells may have been expected. Indeed, we [20] and others [34] have reported increases in basal transamidating activity in cells that were transfected with R580A-TG2. This may be due to methodological differences, as in the current study we used an order of magnitude less of the 5-(biotinamido)pentylamine (BAP) (acyl acceptor in the transamidating reaction) to measure *in situ* transamidating activity since at higher concentrations non-specific background activity increases. Another possible explanation for these seemingly contradictory results would be the fact that guanine nucleotides and Ca^2+^ ions are probably not the only regulators of the transamidating activity of TG2 [3], [12], [20].

It should also be noted that although both the C277S and W241A mutations knock out transamidating activity, there are distinct differences between the two constructs. The former one has been used in numerous studies as a catalytically inactive form of TG2 [1], [19], [35]. However, it is now clear that mutation of C277 does not only abolishes transamidating activity but also significantly reduces guanine nucleotide binding likely due to a significant change in the overall structure of the protein [3], [20]. Therefore, in this study we also used the W241A mutant, which has no detectable effect on GTP binding (Fig. 2), in addition to the C277S mutant, in order to determine whether these two mutations have differential effects on the role of TG2 in cell death. Our data shows that C277S-TG2 and W241A-TG2 do not differ in their ability to protect against most of the stressors suggesting that guanine nucleotide binding is not a major factor. However, there is an exception to this general conclusion. W241A-TG2 significantly improved viability after prolonged treatment with lower concentrations of thapsigargin treatment and serum starvation as measured by the resazurin assay, whereas C277S-TG2 was not protective (see below). It has previously been reported that GTP binding of TG2 in certain paradigms is important for its role in cell survival/death [22]. Our results support the conclusion that guanine nucleotide binding in certain stress conditions may play a role in the protective function of TG2. Overall, these data shows that the prosurvival effect of TG2 is strongest when it has no transamidating activity and efficiently binds to guanine nucleotides. This information might be valuable for any therapeutic approach which might target or exploit the pro-survival function of TG2.

Thapsigargin is a very potent inhibitor of the sarco/endoplasmic reticulum Ca^2+^ ATPase (SERCA) pump and empties ER Ca^2+^ stores while increasing cytosolic Ca^2+^ concentrations. In addition to the increased cytosolic Ca^2+^ effect, it also impairs the ER functioning and triggers the unfolded protein response (UPR) pathway (ER-stress). Given the fact that the concentration of thapsigargin used in HEK 293A cells was 15 μM, the effect of this drug might not be restricted to SERCA inhibition. For instance, in a study conducted on isolated mitochondria, the same concentration of thapsigargin induced maximal mitochondrial swelling and MPTP opening independent of extra-mitochondrial Ca^2+^ concentrations [36], which would result in Ca^2+^ release. Indeed, thapsigargin concentrations used in HEK 293A cells caused a moderate (1.5–2 fold) increase in *in situ* transamidating activity in R580A-TG2 and NLS-R580A-TG2 transfected cells (data not shown) clearly indicating a sustained increase in intracellular Ca^2+^ levels. Intriguingly, LDH release and caspase-3 activity were significantly increased in R580A-TG2 transfected cells but not in NLS-R580A-TG2 transfected cells. This suggests that increased transamidating activity in the cytosol facilitates cell death, but in the case of NLS-R580A-TG2 the amount of active TG2 in the cytosol might not be sufficient to induce cell death since the majority (approximately 80%) is located in the nucleus. Alternatively, increased localization of the protein to the nucleus might be counteracting the detrimental impact of the increase in cytoplasmic transamidating activity. This alternative is especially plausible since it has been shown that nuclear localized transamidating – inactive TG2 protects against apoptosis induced by thapsigargin [28]. The resazurin assay measures the reducing capacity of a viable cell, therefore is sensitive to the redox state of the intracellular milieu. The results of this assay suggests that W241A-TG2 and NLS-W241A-TG2 are protective against thapsigargin-induced cell death in HEK 293A cells, although this protection is not reflected in LDH release and caspase-3 activity assays. The reason of this seemingly contradictory result might be the fact that TG2 has more than one intervention point in the cell death process. Since each assay measures a different aspect of cell death, a multifunctional protein such as TG2 might differentially modulate these different aspects and give contradictory results in different assays.

As mentioned above, 15 μM is a rather high concentration of thapsigargin and thus it is possible that there could be some off target effects; some of which might be relevant to TG2 (such as a possible decrease in the intracellular GTP levels). Therefore we investigated how TG2 impacted cell viability at concentrations of thapsigargin that did not result in significant cell death. To this end, we treated HEK 293A cells with thapsigargin at concentrations of 1 to 5 μM for 24 h. However this treatment resulted in no decrease in cell viability with any assay (data not shown). However, HEK 293TN cells did show a significant loss of cell viability as determined by the resazurin assay when treated with 2.5 μM thapsigargin treatment for 24 h in serum free conditions. Since the concentration of the thapsigargin used was lower (2.5 μM), considerably longer incubation periods were needed (24 h) to see a significant decrease in cell viability, compared to the 9–10 h of treatment with 15 μM thapsigargin in HEK 293A cells. However, it became clear that serum starvation alone significantly attenuated the viability of HEK 293TN cells unlike HEK 293A cells, most probably due to *(i)* increased incubation periods and *(ii)* a greater sensitivity of HEK 293TN cells to stressors. As shown in Fig. 5, cytoplasmic W241A-TG2 significantly improved metabolic activity in HEK 293TN cells as measured by resazurin assay upon serum starvation and serum starvation combined with 2.5 μM thapsigargin, although no effect on LDH release or caspase activity was observed with this treatment paradigm.. Overall, it seems plausible to speculate that W241A-TG2 facilitates cell viability perhaps by improving the metabolic activity of the cell, as indicated by the improvement in the reduction of resazurin and the fact that TG2 has been found associated with mitochondria [37] This role is probably distinct from the more frequently observed protective role of nuclear TG2.

The osmolarity of the bodily fluids are under tight regulation since sustaining isosmolar conditions is essential for the normal functioning of the cells. Under normal physiological conditions, only a small subset of cells is exposed to hyperosmotic stress, such as the endothelial cells of the Bowman's capsule of kidney. However, some pathological conditions, such as ischemia could result in the cells being subjected to osmotic stress [38]. In contrast to the results in human neuroblastoma SH-SY5Y cells stably expressing TG 2 [18], we could not detect an increase in *in situ* transamidating activity in any of our wild type or mutant TG2 transfected cells upon hyperosmotic stress (data not shown). Differences in transamidating activity measurement methodology, cell types and stable overexpression in comparison to transient transfection of TG2 may explain the lack of transamidating activation in response to hyperosmotic stress. Very intriguingly, in that study caspase-3 activation and nuclear condensation were significantly increased in wild-type TG2 cells, compared to C277S-TG2 and vector control cells. However in the current study, caspase-3 activation was not altered by any of the TG2 constructs. Overall, these results suggest that TG2-mediated enhancement of caspase-3 is dependent on its transamidating activity. In contrast to the findings with caspase-3, all of the TG2 constructs, regardless of nuclear or cytoplasmic localization, significantly decreased LDH release in response to hyperosmotic challenge. These results are also in line with that of abovementioned study [18] in which both active and inactive forms of TG2 decreased the LDH release upon sorbitol treatment. These findings argue for a caspase-independent protection mechanism. Similarly, most but not all constructs were protective in the resazurin assay. This small discrepancy between the results of LDH release assay and resazurin assay might be explained by the fact that these assays were performed at different time points. It is also notable that the constructs (R580A and Y516F) that were not protective in the resazurin assay exhibited transamidating activity at lower Ca^2+^ concentrations than wild type-TG2. Although we did not detect any increase in the *in situ* transamidating activity upon sorbitol treatment in R580A-TG2 and Y516F-TG2 transfected cells, it should be noted that the detection limit of the assay is rather modest. An undetectable increase in transamidating activity in R580A-TG2 and Y516F-TG2 transfected cells might counteract the protection.

TG2 protects rat primary cortical neurons and SH-SY5Y neuroblastoma cells against OGD-induced cell death, possibly through its interaction with hypoxia inducible factor 1 beta (HIF1β) and suppression of HIF signaling [9]. Transient transfection of the TG2 constructs did not suppress HIF signaling when expressed as predominantly as a cytosolic protein. However when expressed with an NLS tag, regardless of the mutation, TG2 significantly attenuated HIF signaling in hypoxia. In addition, wild type-TG2, C277S-TG2 and W241A-TG2 constructs protected the cells against OGD significantly only when they were targeted to nucleus. NLS-R580A-TG2 and NLS-Y516F-TG2 constructs, while successfully suppressing HIF signaling, failed to protect cells against OGD. It should be noted that these are the mutations that allow TG2 to be activated at lower Ca^2+^ concentrations and this may be a contributing factor to their diminished ability to protect against OGD. Although no increases in transamidating activity were detected after OGD with any of the constructs (data not shown), as discussed above, it is possible that there was undetectable or substrate specific increase in transamidating activity which could counteract the protection brought by HIF suppression. In addition to this, when TG2 was not targeted to the nucleus, the same TG2 mutants (i.e., wild type-TG2, C277S-TG2 and W241A-TG2) tended to confer protection without reaching statistical significance. These observations all point to the same conclusion: TG2 in the nucleus, most probably through its scaffolding function, suppresses HIF signaling. This suppression can only partially contribute to the protective role of TG2 against OGD induced cell death.

The role of TG2 in cell demise is extremely controversial. There are reports suggesting that transamidating activity of TG2 can be detrimental [18] as well as protective [31]. Other studies have provided evidence that transamidating activity is not important in the context of cell death but it is guanine nucleotide binding activity that determines the role of TG2 in apoptosis [22]. In addition, the protein-protein interactions or scaffolding function of TG2 was shown to be extremely important for cell death-survival decisions, as well [39]. There is also evidence suggesting that the subcellular localization [28] or the type of the stressor [18] determines whether TG2 exacerbates or prevents death. Lastly, it has been reported that in certain stress conditions intronic read throughs of TG2 occur resulting in shortened forms of the protein with unique C-termini [30] which have been reported to be more pro-apoptotic than full length protein [29]. Interestingly, the pro-apoptotic role of these variants might be relevant neither to the transamidating activity nor to the guanine nucleotide binding capacity, but to the fact that they render TG2 more prone to an aggregate forming conformation [29].

**Figure 8:**
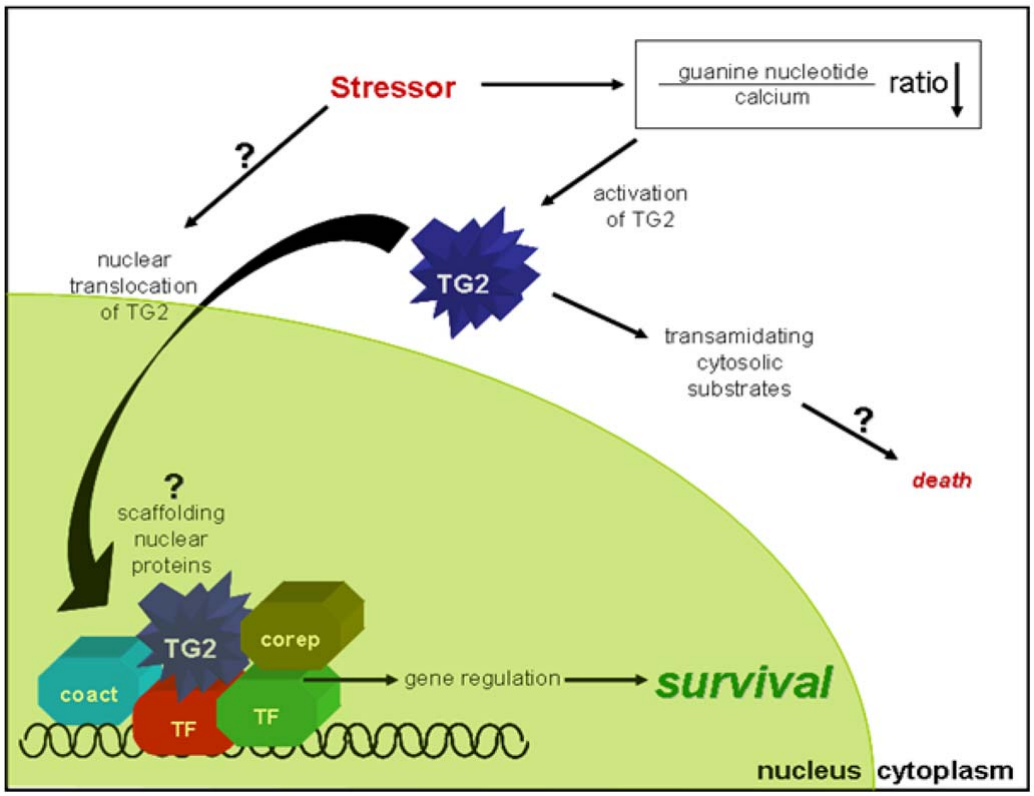
Hypothetical model of the role of TG2 in cell death/survival paradigm. Depending on the stressor type and intensity, TG2 can facilitate or counteract cell death process. Some stressors, such as OGD, trigger nuclear translocation of TG2 through an unknown mechanism, where TG2 mainly promotes survival processes possibly through intervening transcriptional machinery by scaffolding certain transcription factors (TF), co-activators (coact) or co-repressors (corep). Conversely, some stressors dramatically decrease the guanine nucleotide to calcium ratio and elevate transamidating activity of TG2. The increase in the transamidating activity generally means that the death decision has been made and the transamidating function of TG2 helps to execute the death process.

The data in this study clearly show that TG2 can differentially influence the fate of the cell depending on the type of stressor, the activity state, conformation and localization of the enzyme. Furthermore, even in response to the same stressor, TG2 differentially modulates specific aspects of the cell death pathway, even in opposing directions. Due to the multifactorial effect of TG2 on cell death pathways, specific conclusions about the role of this protein in cellular death remain elusive. Nonetheless, by close examination of our results it is possible to extract some tangible generalizations (see Figure 8). Our results support the predominant hypothesis that transamidating activity of TG2 is generally kept very low under resting conditions. The R580A mutation facilitated the pro-apoptotic role of TG2 by allowing it to express transamidating activity in response to certain stressors, as it is usually an inactive and protective protein. Nuclear localization of catalytically active cytosolic TG2 was sufficient to counteract its pro-death role. Similarly nuclear targeting of inactive TG2 variants reinforced their protective effect in most cases. However, in certain cases, cytosolic inactive TG2 which strongly binds guanine nucleotides improved the metabolic profile of the cell more efficiently than the nuclear TG2 when the stress was mild and did not result in overt cell death. Overall, our data strongly suggest that the physiological function of TG2 is likely not primarily as a transamidating enzyme but rather as a scaffolding or adaptor protein, and that only in certain types of extreme stress conditions when death is unavoidable is the transamidating activity increased possibly to facilitate a more organized cell death process. Otherwise, TG2 is mostly protective.

## Materials and Methods

### Constructs

The untagged TG2 constructs were cloned into pcDNA 3.1 (+) (Invitrogen); pShooter pCMV/nuc/myc vector (Invitrogen) was used to make the NLS-tagged TG2 constructs. Untagged wild type-TG2, C277S-TG2 and R580A-TG2 have been described previously [20], [40]. W241A-TG2 and Y516F-TG2 were prepared for this study using the pcDNA-wildtype-TG2 construct as a template and the GeneEditorTM in vitro Site-Directed Mutagenesis System (Promega). The primer used to make the W241A-TG2 construct was 5′- GCT GGG ACG CGC GGA CAA CAA CTA C- 3′ and for the Y516F-TG2 construct was 5′- CTG TGC CCG CAC CGT CAG CTT CAA TGG GAT CTT GG- 3′. Mutagenesis was carried out according to the manufacturer's instructions and the presence of the mutations was confirmed by sequencing. NLS tagged constructs were prepared as described previously [28]. The resulting NLS-tagged constructs were verified by sequencing.

### Cell Culture

HEK 293A cells were cultured in Dulbecco's modified Eagle's medium (Irvine Scientific) supplemented with 5% fetal bovine serum (HyClone), 2 mM L-glutamine (Invitrogen, Life Technologies, Inc.), 100 µg/ml streptomycin (Invitrogen, Life Technologies, Inc.) and 100 units/ml penicillin (Invitrogen, Life Technologies, Inc.). For HEK 293TN cells, the plates were coated with 0.1 mg/mL poly-L-lysine in PBS 24 h prior to use. The plates were rinsed with PBS once immediately prior to use and the cells were cultured on these plates in Dulbecco's modified Eagle's medium/F12 medium (50∶50) (Irvine Scientific) supplemented with 5% bovine growth serum (HyClone), 2 mM L-glutamine, 100 µg/ml streptomycin and 100 units/ml penicillin. Cells were grown in a humidified atmosphere containing 5% CO_2_ at 37°C. Transient transfections were carried out using FuGENE 6 reagent (Roche Applied Science) or Lipofectamine 2000 (Invitrogen) according to the manufacturers' instructions.

### Cell Treatment Paradigm

24 h after transfection, cells were transferred to serum-free media in which the treatments performed. For thapsigargin treatment, HEK 293A cells were treated with 15 µM thapsigargin (Alexis) in serum-free medium for 10 h (LDH release assay) or 6 h (caspase-3 and resazurin assay) at 37°C. HEK 293TN cells were treated with 2.5 µM thapsigargin in serum-free medium for 24 h. The thapsigargin was prepared in Me_2_SO and the control cells were treated with Me_2_SO under the same conditions. For hyperosmotic stress, 1.0 M sorbitol solution was prepared in serum free media and the HEK 293A cells were treated in sorbitol/media solution for 9 h (LDH release assay) or 6 h (caspase-3 and resazurin assay) at 37°C. Control cells were treated in serum free media under the same conditions. For oxygen glucose deprivation, HEK 293A cells were transferred to serum and glucose free Dulbecco's modified Eagle's medium (Irvine Scientific) and incubated in a humidified atmosphere containing 5% CO_2_ and 0.1% oxygen at 37°C for 16 h. Control cells were treated in serum free glucose containing normal DMEM in a humidified atmosphere containing 5% CO_2_ and ambient oxygen at 37°C. For the HRE luciferase reporter assay cells were transferred to serum free media and incubated in a humidified atmosphere containing 5% CO_2_ and 0.1% oxygen at 37°C for 16 h. Immediately after the incubation the activity of the reporter was measured. Control cells were treated in serum free media in a humidified atmosphere containing 5% CO_2_ and ambient oxygen at 37°C.

### Nuclear Fractionation

Nuclear fractionation studies were conducted as previously described [28] with slight modifications. Briefly, HEK 293A cells were washed twice, harvested in ice cold PBS and the cell pellets were resuspended in lysis buffer (10 mM Tris, pH 7.5, 10 mM NaCl, 3 mM MgCl_2_, 0.05% Nonidet P-40, 1 mM EGTA) with protease inhibitors (0.1 mM phenylmethylsulfonyl fluoride, and 10 µg/ml of each of aprotinin, leupeptin, pepstatin A) by triturating followed by centrifugation at 380 ×g for 5 min at 4°C. The supernatants were collected and used as the cytosolic fractions. The pellets were washed once in lysis buffer and twice in wash buffer (30 mM sucrose, 10 mM Pipes, pH 6.8, 3 mM MgCl_2_, 1 mM EGTA, 25 mM NaCl) with protease inhibitors. The crude nuclei were overlaid on the top of 0.7 M sucrose with protease inhibitors, and spun at 1200 ×g for 10 min at 4°C. The pellets were collected and resuspended in buffer B (300 mM sucrose, 10 mM Pipes, pH 6.8, 3 mM MgCl_2_, 1 mM EGTA, 25 mM NaCl, 0.5% Triton X-100) with protease inhibitors and used as the nuclear fractions. The proteins were visualized by immunoblot analysis.

### Immunoblotting

Cells were rinsed in ice-cold phosphate-buffered saline (PBS) and collected in lysis buffer, containing 0.5% NP-40, 150 mM NaCl, 10 mM Tris-Cl (pH 7.4), 1 mM EGTA, 1 mM EDTA, 1 mM phenylmethylsulphonyl fluoride, 1 µM okadaic acid, and 10 µg/ml each of aprotinin, leupeptin, and pepstatin. Samples were sonicated on ice and centrifuged at 16,000 g for 10 min. Protein concentrations of supernatants were then determined by the bicinchoninic acid assay with bovine serum albumin (BSA) as a standard and samples were diluted to a final concentration of 1 mg/ml with 2× reducing stop buffer (0.25 M Tris-HCl, pH 6.8, 5 mM EDTA, 5 mM EGTA, 25 mM dithiothreitol, 2% SDS, 10% glycerol, and bromophenol blue as the tracking dye). Samples (5–25 µg of protein depending on the assay) were resolved on 10% SDS-polyacrylamide gels, and transferred to nitrocellulose. Blots were blocked in 5% nonfat dry milk in TBST (20 mM Tris-HCl, pH 7.6, 137 mM NaCl, 0.05% Tween 20) for 1 h at room temperature. The blots were then incubated overnight with a mouse monoclonal TG2 primary antibody TG100 or CUB7402 (Lab VisionNeoMarkers, Fremont, CA, USA; 1∶5,000 dilutions) The membranes were then washed three times with TBST and incubated with HRP-conjugated secondary antibody for 1 h at room temperature. The membranes were rinsed three times for 30 min with TBST, followed by four quick rinses with distilled water, and developed with the enhanced chemiluminescence as described previously [41].

### GTP-agarose pull-down assay

GTP-agarose pull-down assay was performed according to the procedure previously described [33] with slight modifications. Briefly, 24 h after transfection, HEK 293A cells were rinsed in ice-cold PBS and collected in GTP-binding buffer (20 mM Tris-HCl pH:7.5, 5 mM MgCl2, 2 mM PMSF, 20 µg/mL leupeptin, 20 µg/mL pepstatin, 10 µg/mL aprotinin plus 150 mM NaCl and 0.1% Triton-X [for low stringency] or 300 mM NaCl and 0.5% Triton-X [for high stringency]). Samples were sonicated for 15 s and centrifuged at 13,000 g for 10 min at 4°C, and the supernatant was collected. The protein concentration of each supernatant was determined by the BCA assay. 100 µg of lysate protein were incubated with 100 µL of GTP-agarose beads (Sigma-Aldrich; equilibrated in GTP-binding buffer) in a total of 500 µL of GTP-binding buffer for 30 min at 4°C. The beads were centrifuged at 10,000 g for 2 min and the supernatant was retained. Then, the beads were washed three times with 1 ml of GTP-binding buffer and the retained supernatant was incubated with the beads for another 30 min. The beads were washed again as described above and then incubated with the retained supernatant overnight at 4°C. After washing seven times with GTP-binding buffer, bound protein was eluted from the beads by boiling them in 50 µL of 2× reducing stop buffer. TG2 that had bound to the GTP-agarose beads was visualized by performing immunoblot analysis on the eluted protein.

### In vitro Transglutaminase Assay

*In vitro* transamidating activity measurement was conducted as described previously [18] with slight modifications. Briefly, 24 h after transfection, HEK 293A cells were washed with ice-cold PBS and harvested in lysis buffer which is composed of 50 mM Tris-HCl (pH 7.5), 0.25 M sucrose, 1 mM EDTA, 0.1 mM phenylmethylsulfonyl fluoride, and each of aprotinin, leupeptin, and pepstatin at a concentration of 10 µg/ml. Samples were briefly sonicated on ice, spun at 10,000 g and 4°C for 1 min. Supernatant was transferred to another tube and protein concentration of the supernatant was determined using the BCA assay. Samples were diluted to a final concentration of 2.5 mg/ml with lysis buffer. Samples containing 250 µg protein was incubated in transamidating assay buffer containing 0.1 M Tris-HCl (pH 7.5), 10 mM dithiothreitol, 2.5% Lubrol, 0.2 mM putrescine (nonradioactive), 1 µCi of [1,4(n)-3H]putrescine dihydrochloride, 3.0 mg/ml N,N-dimethylcasein, 0.25 mM GTPγS and given amount of CaCl_2_. Reaction mixtures were incubated for 1 h at 37°C, and the reaction was terminated by addition of trichloroacetic acid to a final concentration of 10% (w/v). The samples were incubated on ice for 1 h and centrifuged at 16,000 g and 4°C for 20 min. The supernatant was removed, and the pellet was rinsed twice with 1 ml of 5% (w/v) trichloroacetic acid, then 250 µL of 0.25 M NaOH was added to each tube. The samples were incubated at 100°C for 10 minutes, or until the pellet is completely dissolved, by intermittent and vigorous mixing. The radioactivity emitted from bound [3H]putrescine was quantitated by liquid scintillation using a Beckman LS6500 scintillation counter, and TG activity was calculated after background subtraction as fold increase over the radioactive reading obtained from the lysate of empty vector transfected cells.

### In situ Transglutaminase Assay

*In situ* transamidating activity measurement was conducted as described previously [42] with slight modifications. Briefly, 24 h after transfection of HEK 293A cells, normal growth media was changed to fresh growth media with 5% FBS containing given amount of ionomycin (0–1.5 µM) and 0.1 mM 5-(biotinamido)pentylamine (BAP) (Pierce). 3 h later, cells were collected in media, pelleted, washed once with PBS and resuspended in homogenization buffer (50 mM Tris-Cl, pH: 7.5, 150 mM NaCl, 1 mM EDTA), sonicated on ice. In a high protein binding 96-well plate (Falcon), 10 µg of protein was loaded in a total of 50 µL homogenization buffer and the plate was incubated at 4°C overnight. The next day, 200 µL of blocking buffer (5% BSA, 0.01% Tween 20 in borate saline [100 mM boric acid, 20 mM Na-borate and 0.76 mM NaCl]) was added to each sample and the plate was incubated at 37°C for 1 h. Each well was then rinsed 3 times with rinsing buffer (1% BSA, 0.01% Tween 20 in borate saline) and incubated with 1 µg/µL Horseradish peroxidase conjugated neutravidin (Pierce) in a total of 100 µL rinsing buffer at room temperature for 1 h. The samples were washed 4 times with rinsing buffer, and peroxidase reaction was conducted in the dark in 200 µL of OPD buffer (0.1 M Na_2_HPO_4_, 50 mM citric acid, 1 tablet o-phenylenediamine (Sigma Aldrich), 0.0006% H_2_O_2_). After 20 minutes, the reaction was stopped by addition of 50 µL of 3 M HCl and read at 493 nm wavelength.

### Caspase-3 activity assay

Caspase-3 activity was measured using a fluorometric assay. Cells were plated in 60 mm plates and treatments were performed as described above. Immediately after the treatment, cells were rinsed once with PBS and harvested in lysis buffer (20 mM Tris, pH 7.5, 150 mM NaCl, 2 mM EDTA, 2 mM EGTA, 0.5% NP40, 0.1 mM PMSF, and a 10 µg/mL final concentration of each of aprotinin, leupeptin, and pepstatin). Samples were sonicated for 15 s and centrifuged at 13,000 rpm for 10 min at 4°C, and the supernatant was collected. The protein concentration of each supernatant was determined by the BCA assay and the samples were diluted to 1 µg/µL. Cell lysates (20 µg) were incubated for 1 h at 37°C, in 200 µL of reaction buffer [(20 mM HEPES, pH 7.5, 10% glycerol, 2 mM dithiothreitol (DTT)], containing caspase-3 substrate (Ac-DEVD-AMC) (Alexis Biochemicals, San Diego, CA, USA) at a concentration of 25 ng/µL (Bijur et al. 2000). Fluorescence was measured using a fluorescence plate reader (BioTek Synergy HT Multi-Detection Microplate Reader) at wavelengths, excitation 360 nm and emission 460 nm.

### Lactate dehydrogenase (LDH) release assay

LDH release was measured using an LDH release assay kit (Roche). Cells were plated in 24-well plates and treatments were performed as described above. After the treatment was finished, media and the cell lysates were collected and assay was performed to assess cell viability. LDH release was measured as described previously [18].

### Resazurin Assay

Resorufin production by viable cells was determined by CellTiter-Blue Cell Viability Assay kit (Promega). Cells were plated in 24-well plates and treatments were performed as described above. At the end of the treatments, resazurin was added to the media according to manufacturer's instructions and incubation was continued for 1 h. At the end of the incubation, resorufin fluorescence was measured using a fluorescence plate reader (BioTek Synergy HT Multi-Detection Microplate Reader) at wavelengths, excitation 540 nm and emission 590 nm. Results are presented as a percent of control cells.

### HRE luciferase reporter assay

HEK 293A cells were plated in a 24-well plate and transiently transfected with TG2 constructs together with a *firefly* luciferase vector under control of HRE-bearing 64-mer fragment from human *enolase* promoter [43] and *Renilla* luciferase vector [9] using Fugene 6 reagent as described above. 24 h post-transfection, cells were transferred to serum free media and placed in a hypoxic environment for 16 h at 37°C as described above. Control cells were kept in ambient oxygen concentrations under the same conditions. Luciferase activity was measured in cellular lysates using the Dual-Luciferase Reporter Assay System Kit (Promega, Madison, WI, USA) according to the manufacturer's protocol and a TD-20/20 Luminometer. For each sample, the Firefly luciferase data was normalized to the Renilla luciferase internal control.
